# Culture Linked to Increasing Ageism during Covid-19: Evidence from a 10-billion-word Corpus across 20 Countries

**DOI:** 10.1093/geronb/gbab057

**Published:** 2021-03-31

**Authors:** Reuben Ng, Ting Yu Joanne Chow, Wenshu Yang

**Affiliations:** 1 Lee Kuan Yew School of Public Policy, National University of Singapore, Singapore; 2 Lloyd’s Register Institute for the Public Understanding of Risk, National University of Singapore, Singapore

**Keywords:** Aging narratives, age stereotypes, pandemic, psychomics, text as data, quantitative social science

## Abstract

**Objectives:**

Older adults experience higher risks of getting severely ill from COVID-19, resulting in widespread narratives of frailty and vulnerability. We test: (1) Whether global aging narratives have become more negative from before to during the pandemic (Oct’19 to May’20) across 20 countries; (2) Model pandemic (incidence and mortality), and cultural factors associated with the trajectory of aging narratives.

**Methods:**

We leveraged a 10-billion-word online-media corpus, consisting of 28 million newspaper and magazine articles across 20 countries, to identify nine common synonyms of ‘older adults’ and compiled their most frequently-used descriptors (collocates) from Oct’19 to May’20—culminating in 11,504 collocates that were rated to create a Cumulative-Aging-Narrative-Score-(CANS) per month. Widely used cultural dimension scores were taken from Hofstede, and pandemic variables, from the Oxford COVID-19 Government Response Tracker.

**Results:**

Aging narratives became more negative as the pandemic worsened across 20 countries. Globally, scores were trending neutral from Oct’19 to Feb’20, and plummeted in Mar’20, reflecting COVID-19’s severity. Pre-pandemic (Oct’19), UK evidenced the most negative aging narratives; peak-pandemic (May’20), South Africa took on the dubious honor. Across the 8-month period, Philippines experienced the steepest trend towards negativity in aging narratives. Ageism, during the pandemic, was ironically, not predicted by COVID-19’s incidence and mortality rates, but by cultural variables: Individualism, Masculinity, Uncertainty Avoidance, and Long-term Orientation.

**Discussion:**

The strategy to reverse this trajectory lay in the same phenomenon that promoted it: A sustained global campaign—though, it should be culturally nuanced and customized to a country’s context.

Ageism is linked to poorer health, higher mortality, and costs the US healthcare system $63 billion/year (Levy, et al., 2020). It is defined as the discrimination of older adults based on their age (Butler, 1969), and expanded by scholars to include negative portrayals of older adults as derived from age-based stereotypes centered on illness, irrelevance, and incompetence (North & Fiske, 2015). Recently, 194 member states called on the World Health Organization (WHO) to develop a global campaign to combat ageism citing its insidious threat to health (Officer & de la Fuente-Núñez, 2018). This remarkable global consensus—rare in a period of peak polarization—is an awakening to ageism's consequences on population health and its alarming prevalence in health policy (Lloyd-Sherlock, Ebrahim, McKee, & Prince, 2016).

The "ageism creep" into global public policy is unlikely a product of intention but a lack of awareness: Ageism is the least acknowledged discrimination among other forms, such as sexism and racism, and is prevalent in at least three ways. One, until 2010, disability adjusted life years (DALYs) applied age-biased weighting where a year's loss of life in old age is worth less than those at a younger age—specifically, a year lived at 75 years is valued less than half of a year lived at 35 years (Arnesen & Kapiriri, 2004). Two, WHO's five-year action plan for the prevention of non-communicable diseases (NCDs) highlighted gender, ethnicity, and persons-with-disabilities, but not old age (WHO, 2008). The action plan targeted individuals aged 25 to 64 years for key NCD indicators such high blood pressure, but no evidence or explanations were provided for these arbitrary age limits. Three, population-wide research studies tend to provide selections of five-year age bands up to 64 years, with "65 years and above" as a final catch-all category (Little, 2018). This is ludicrous given the diversity of needs and characteristics of the young-old (65-74 years), middle-old (75-85years) and old-old (85 years and above).

Unfortunately, the COVID-19 pandemic has posed a significant threat to older adults who evidenced higher risk of getting severely ill or dying from the virus. Mortality rates in China increased with age, with those above 60 years evidencing a death rate of over 10%, compared to 0.66% for all age groups (Verity et al., 2020). European nations estimated that 95% of all deaths were from those above 60 years (World Health Organization Regional Office for Europe, 2020). Inexorably, public communications and journalism have directed extra attention to older adults, highlighting their heightened risk as causes for concern. For instance, the Singapore government (2020) buys a daily advertorial in the front page of the national English newspaper warning that "…in Singapore, the majority who have died due to COVID-19 have been seniors" so families should keep older adults at home and help them with essential needs. Other governments have issued similar public communications (e.g., New Zealand Government, 2020; Government of Canada, 2020). These laudable efforts are well-intended, though most of them, unbeknownst to their creators, contain varying shades of ageism. Ayalon and colleagues (2020) noted that most news articles about older adults during the pandemic have portrayed them as helpless and frail, amidst increasing prevalence of disability (Ng, Lim, Saw, & Tan, 2020); Monahan and colleagues (2020) posited that well-intentioned public health responses meant to protect seniors may inadvertently intensify negative stereotyping of older adults as weak.

Against this background, our paper aims to achieve two objectives—and test two hypotheses. First, aging narratives have become more negative as the pandemic unfolds from October 2019 to May 2020 (Hypothesis 1). We started our analysis in October 2019 to ascertain baseline ageism during the pre-pandemic phase and observe its trajectory as the pandemic progressed, across 20 countries, using a 10-billion-word corpus of over 7,000 online newspapers and magazines—the largest cross-cultural corpus of online media (Davies, 2017). The 20 countries span North America, Europe, Africa, Asia, and Oceania, presenting a comprehensive global perspective of aging narratives from before to during the pandemic. According to Cultivation Theory (Gerbner, Gross, Morgan & Signorielli, 1994), the large representation of online media within the corpus reflects societal perceptions of respective countries and provide an extraordinary platform to study aging narratives.

Our empirical study of global ageism during COVID-19, across 20 countries, builds on recent work, albeit mostly commentaries. For example, Meisner (2020) observed that the generalization of age-related biomedical risks led to the exacerbation of ageism on social media. An analysis of sample tweets in March 2020 suggested that approximately 25% of tweets about Covid-19 and older adults contained ageist or potentially offensive content (Jimenez- Sotomayor et al., 2020). The trend of grouping older adults as a homogenously frail group, vulnerable to Covid-19, is criticized as a form of benevolent ageism (Koepp, 2020), as it overemphasizes the image that older adults are helpless 'sitting ducks' (Fraser et al, 2020), and glosses over how older adults are diverse and capable contributors of society (APA, 2020). The narrative of frailty during Covid-19 has observably led to increased feelings of heightened loneliness and worthlessness (Brooke & Jackson, 2020).

Our second objective is to analyze factors associated with the (hypothesized) negativity of aging narratives during the pandemic. Most commentaries have settled on the most expedient conclusion: Pandemic-related attributes such as the incidence and mortality rates are driving the negativity of aging narratives (pandemic hypothesis). While intuitive, we argue that ageism is more deep-seated— it has been increasing over 200 years (Ng, et al., 2015; Ng & Chow, 2020), and studies found that cultural factors are associated with ageism after adjusting for demographics (Ng & Lim, 2020). We hypothesize that both pandemic variables (Covid-19 incidence and mortality rates) and cultural variables (measured by Hofstede's cultural dimensions) are associated with aging narratives from pre-to-peak pandemic (Hypothesis 2).

We selected Hofstede's five dimensions (2001) as they are one of the most widely used measures of cultural values. Though these measures are criticized as often as they are cited, the Hofstede measures remain appropriate to this study for three reasons. First, they provide multiple dimensions from which a culture can be understood. Second, they are widely recognized and understood, demonstrating our study's comparability and contribution to prior literature. Third, they are based on a national-level understanding of culture, which has been shown to differ from individual-level culture (Zhang et al., 2016), and therefore compatible with the scope of our study as we observe aging narrative at the national level across 20 countries. Further, recent studies show the link between Hofstede's dimensions and ageism (Ng & Lim, 2020).

The five cultural dimensions, defined in Hofstede (2011), are as follows: *Individualism* is "the degree to which individuals are supposed to look after themselves or remain integrated into groups, usually around the family"; *Masculinity* is "the distribution of emotional roles between the genders (…); it opposes 'tough' masculine to 'tender' feminine societies"; *Uncertainty Avoidance* is "the extent to which a culture programs its members to feel either uncomfortable or comfortable in unstructured situations"; *Long-term Orientation* is "the extent to which a culture programs its members to accept delated gratification of their material, social, and emotional needs"; *Power Distance* is "the extent to which the less powerful members of organizations and institutions accept and expect that power is distributed unequally."

Overall, our study is significant is three ways. Conceptually, we contribute to societal ageism by analyzing how an extraordinary event, such as the COVID-19 pandemic, may dynamically influence aging narratives. Second, we extend the field of cultural gerontology by examining the impact of culture on aging narratives across 20 countries during a pandemic. Practically, understanding the cultural underpinnings of ageism lay the groundwork for designing policy interventions to reduce it, as studies show the malleability of cultural frames (Dweck, Chiu & Hong, 1995)—our study provides the cultural considerations to do this effectively.

### Methods

#### Dataset

The News on the Web corpus (Davies, 2017) is the largest cross-cultural corpus that consists of over 7000 online newspapers and magazines with over 28 million articles across 20 countries. The corpus is dynamic, with 200 million new words, from 300,000 new articles, added every month. This dataset was created with funding from the National Science Foundation (NSF) and the National Endowment for the Humanities (NEH) to study contemporary language usage in countries where English is widely used. Cultivation Theory (Gerbner, Gross, Morgan & Signorielli, 1994) suggests that the large representation of online media reflects the societal perceptions of respective countries and provides an extraordinary platform to study aging narratives. The countries span six regions: North America (America, Canada); Europe (Ireland, United Kingdom); Oceania (Australia, New Zealand); Asia (Bangladesh, Hong Kong, India, Malaysia, Pakistan, Philippines, Singapore, Sri Lanka); Africa (Ghana, Kenya, Nigeria, South Africa, Tanzania); The Caribbean (Jamaica). Within the study's timeframe (October 2019 to May 2020), our corpus consists of 1.5 billion words.

#### Measurement of Aging Narratives

Following previous studies (e.g., Ng et al., 2015), we shortlisted the most common synonyms of an older adult: *elder(s), elderly, seniors, old(er) adult(s), old people, old age, senior citizen(s), (the) aged, nursing home resident*. Based on this list of synonyms, we generated collocates (i.e. words that co-occurred most frequently with each synonym) for each of the 20 countries, every month, between October 2019 to May 2020. This time-frame's start point was selected to account for pre-pandemic representation of older adults two months before the virus was first reported in December 2019; the May endpoint was determined by data availability at the time of analysis. These collocates had the following qualifying criteria: (a) Lexical Proximity: collocate present within six words prior or after the respective synonym; (b) Relevant context: collocate referred to specifically to an old person (checked by two raters); (c) Mutual Information Score of three and above: collocate had a stronger association with the respective synonym compared to other words in the corpus for that country, indicating semantic bonding (Church & Hanks, 1990). This is an application of computational linguistics to study language shifts and used to identify stereotypes in other studies (Ng et al., 2015)—known as Psychomics (Ng & Chow, 2020) . The rigorous process culminated in 11,504 collocates over eight months, across 20 countries.

Thereafter, each collocate that met the study criteria was rated on a scale from 1 (very negative) to 5 (very positive) by two independent raters using Cronbach's alpha was .937 (95% CI: .906, .957) for the scoring method that was validated to measure age-stereotype associated words (Levy & Langer, 1994). For instance, *abuse, suffering* were rated 1 (very negative); *transportation, occupational* were rated 3 (neutral); *venerable, jovial* were rated 5 (very positive). We calculated a Cumulative Aging Narrative Score (CANS) per month by taking the respective mean ratings globally, and by country, to test Hypotheses 1 and 2, respectively.

**Pandemic Variables**

The monthly incidence and mortality rates across 20 countries were derived from the Oxford Covid-19 Government Response Tracker (Hale et al., 2020).

**Hofstede's Five Cultural Dimensions**

Calculations of the country dimension scores are found in Hofstede (2001), and Hofstede and Minkov (2013) that were based on original cross-national surveys of IBM employees, and subsequent studies (e.g., Hofstede Insights, 2020). For the purposes of this study, we have used the country dimension scores obtained from the latter source, as they present the most updated country scores. The country score for each of Hofstede's dimensions was calculated as follows. First, individual survey responses to each question were calculated at the national level. For questions that were answered on a five-point Likert scale, the national mean of the responses was calculated. For questions requiring a Yes/No or multiple-choice answer, the national percentage of each answer, or a combination of answers (e.g. "Option A OR Option C") was calculated. Next, these national-level scores were combined using a weighted formula to yield a country dimension score based on three to eight survey questions, resulting in final scores that ranged from 0 to 100.

**Analytic Strategy**

Hypothesis 1—aging narratives have become more negative as the pandemic deepens from October 2019 to May 2020—was tested using a trend model to show statistically significant linear trends in the positivity/negativity ratings of aging narratives each month and across 20 countries, where higher Cumulative Aging Narrative Score (CANS) indicate more positive age stereotypes, and lower CANS indicate more negative stereotypes. Hypothesis 2— pandemic variables (Covid-19 incidence and mortality rates) and cultural variables (Hofstede's cultural dimensions) are associated with aging narratives from pre-to-peak pandemic—was tested using a mixed model. All data pre-processing, text analytics and statistical analyses were conducted in Python 3.7 and OriginPro 2019b.

## Results

### Aging Narrative Scores across 20 countries during Covid-19

Globally, we found a significant declining linear trend for aging narratives ($\beta$ = -.0211, $p$ < .05), supporting Hypothesis 1 (see Figure 1). Other trends such as quadratic ($\beta$ = .000929, $p$ =.753) and cubic ($\beta$ = .00165, $p$ = .316) were non-significant. Cumulative Aging Narrative Scores (CANS) were trending neutral from Oct'19 to Feb'20, and dived sharply in Mar'20. To analyze the abrupt drop in CANS, we used Grubb's and Dixon's Q—analyses to detect outliers. Across the 8-month period, the Grubb ($G$ = 2.171, $p$ < .05) and Dixon's ($Q$= 0.725, $p$ < .05) suggested that the notable drop in CANS—denoting that aging narratives have become less positive and trending toward negativity—is statistically significant. At the country-level, during the pre-pandemic phase (Oct'19), UK evidenced the most negative aging narratives; peak-pandemic (May'20), South Africa took on the dubious honor. Across the 8-month period, Philippines experienced the steepest trend towards negativity in aging narratives, while Ghana experienced the least steep trend.

### Predictors of Aging Narrative Trends during Covid-19

We tested Hypothesis 2 progressively across two mixed models with pandemic variables (incidence and mortality rates) and cultural dimensions as fixed variables and time as the random variable. Models 1 tested the pandemic hypothesis and Model 2 tested both the pandemic and cultural hypotheses (see Table 1). The pandemic variables of incidence and mortality rates were non-significant while four of five cultural dimensions reached significance: Higher individualism ($\beta$ = -.00141, $p$ < .05), masculinity ($\beta$ = -.00236, $p$ < .001), uncertainty avoidance ($\beta$ = -.00281, $p$ < .001), long-term orientation ($\beta$ = -.00248, $p$ < .001) are associated with increasing negativity of aging narratives, controlling for Covid-19 incidence and mortality rates—providing support for Hypothesis 2. Power distance did not reach significance. There was no evidence for multicollinearity as the variance inflation factors (VIF) scores were below the stringent criteria of five (Sheather, 2009).

### Discussion

This is one of the first known studies to dynamically track global ageism from before to during the COVID-19 pandemic across 20 countries. The finding is consistent with many commentaries, though no less alarming: Aging narratives have become more negative as the pandemic progressed across all 20 countries. Globally, scores were trending neutral from October 2019 to February 2020 and experienced a precipitous drop in March 2020. The neutrality of aging narratives before the pandemic (October'19) is a welcome finding—suggesting a 'boomer dividend'—and addresses the controversy of whether age stereotypes have become more positive or negative as the baby boomer cohort enters old age, following 210 years of increasing negativity in societal sentiments toward older adults (Ng & Chow, 2020). However, the 'boomer dividend' was unfortunately erased by the pandemic in March 2020. Thereafter, aging narrative scores seem to be inching upwards though it has not recovered to pre-pandemic levels.

These insights are significant and contribute to research on aging narratives. Specifically, the 'boomer dividend' accumulated in recent decades can be erased by an extraordinary event, such as COVID-19, that have featured older adults prominently and globally, albeit negatively. It is remarkable that age stereotypes, influenced by deep-seated demographic drivers, could change dynamically in months—and our study has shown the circumstances surrounding this phenomenon. On a positive note, this could mean that a campaign to counter negative sentiments toward older adults could be effective if it is global, prominent, and sustained over a period of time.

Ironically, ageism during the pandemic was not predicted by pandemic statistics (incidence and mortality) but by cultural variables across 20 countries. There are two explanations. First, the pandemic statistics were age-invariant—they were country-level incidence and mortality rates. Age-specific variables such as “number of new cases of older adults aged 60-65/ 66-70/ 70-75” would provide a more nuanced perspective that could elicit age-focused patterns linked to ageism. These statistics were not publicly available across 20 countries and future studies should consider them.

The second explanation is that ageism is deep-seated, and predicated on structural undercurrents of culture, demographics, and societal norms. To this end, we found that four of five cultural dimensions are linked to ageism during the pandemic—individualism, masculinity, uncertainty avoidance, long-term orientation. This is the first known study linking culture and ageism during COVID-19, and contributes significantly to cultural gerontology. We attempt to explain the respective cultural variables linked to ageism.

Individualism was associated with increasing negativity of aging narratives, controlling for covariates. Societies with high individualism scores are linked to the notion that social problems should be dealt with by the individual. Löckenhoff et al. (2009) found that individualistic attitudes are associated with more negative age stereotypes; Estes (1986) found that individualism contributes to reduced support for public programs for the aged, as this ideology differentiates between the “deserving elderly” and “undeserving elderly”. During the pandemic, with older adults being portrayed as requiring additional assistance, this may exacerbate resentment and negative sentiments in societies with higher individualism scores.

Masculinity was associated with increasing negativity of aging narratives, controlling for covariates. Societies that score high on this dimension prioritize 'masculine' traits like toughness and strength, rather than relying on others: Addis (2003) found that men were less likely to seek help for health issues, primarily because of being socialized in backgrounds that prized masculinity. Tannenbaum & Frank (2011) found that older men tended to view their health through a masculine lens: prioritizing resilience and being unfazed by illness. Thus, during a pandemic, societies scoring high on the masculine dimension may further sideline and marginalize the vulnerable for their frailty, or feel less sympathetic towards the weak—thus, expediting increasing negativity in sentiment toward older adults.

Uncertainty avoidance was associated with increasing negativity of aging narratives, controlling for covariates. Societies with high uncertainty avoidance scores tend to be uncomfortable with the unpredictable: Löckenhoff et al. (2009) and Lawrie et al. (2019) found that people from cultures with higher uncertainty avoidance had more negative perceptions of aging. In uncertainty-avoidant cultures, individuals tend to hold negative stereotypes about old age due to heightened aging-related anxieties (Ramírez & Palacios-Espinosa, 2016), which are unavoidable since uncertainty is an inherent part of old age (Ågren, 1998), and associated with unpredictable levels of diminished health and the loss of loved ones (Baltes & Smith, 2003). Thus, societies that prioritize 'certainty' may have a generally negative outlook on the future with the specter of the virus still raging, and vaccination efforts that are behind schedule (as of the study period). This may lead to more negative aging narratives, sparking worry about the coping capabilities of the healthcare system—older adults may be viewed as a liability in the context of the pandemic.

Long-term orientation was associated with increasing negativity of aging narratives, controlling for covariates. Societies that score high on this dimension focus on the future and plan ahead, tending to value people who can contribute the most in the future; thus, prioritizing the young and economically viable. As such, they may be less respectful of people with a shorter 'shelf-life' in the workplace. Furthermore, long-term orientation involves the delay of current gratification for future benefits— security actions (Aurigemma & Mattson, 2019), innovation (Bukowski & Rudnicki, 2019), and resilience (Sulphey, 2020)— which is unfortunately unrealistic in aging societies who have to deal proactively with the needs of the aging population *immediately* or risk spending more for acute care later. During a pandemic, such societies are more likely to resent the reliance that retired older adults have on the economically active population, and in burdening the country's long-term healthcare resources.

The country-specific impact of COVID-19 on ageism is also interesting. The finding that UK evidenced the highest ageism scores amongst the 20 countries corroborated with previous studies of culture and ageism (e.g., Ng & Lim, 2020). During the pandemic in May 2020, South Africa scored highest in societal ageism—this is a novel and intriguing finding. Existing literature point toward two reasons. One, a rapid qualitative assessment of 60 key informants found a heightened awareness that older adults are at higher risk of COVID-19, though false information from social media may have been linked to exaggeration and othering—in this example, the labeling of older adults as the outgroup and the perception that the latter group is a threat (Schmidt et al., 2020). Two, South Africa has been consistently ranked as one of the fast-aging societies in the world with the proportion of older adults increasing rapidly. A scenario-based study found that South African participants expressed surprise and negative sentiments when told of the exponential increase in the older adult population by 2030 (Roux & Viljoen, 2018). Though it is beyond the scope of our paper to delve into explanations for each country, the South African example show that cultural tendencies for ageism (Ng & Lim, 2020) could be exacerbated by a pandemic—an interesting avenue for future studies.

Of broader significance, our findings blunt the deterministic nature of ageism during the pandemic. While COVID-19 has decreased the positive aging narratives accrued by baby boomers, that is only one side of the ageism coin. Our study provides evidence that the cultural side is equally, if not more, important—especially, in the quest for societal solutions to decrease it. The train has left the station on the pandemic reality: There are limited options to change the trajectory of COVID-19 and most governments are doing their best to keep incidence and mortality rates down. Instead, cultural mindsets are more malleable, as priming studies have shown (e.g., Dweck, Chiu & Hong, 1995), and individual-level interventions to decrease interpersonal ageism have achieved considerable success (Lytle, Nowacek, & Levy, 2020). These individual-level efficacy trials need to be scaled up at the societal level, and our study provides the cultural considerations to do this effectively.

Drilling down to societal interventions that decrease ageism, future studies could focus on factors that mediate the culture-ageism relationship. In addition, societal-level interventions could achieve a multiplier effect by targeting countries high in individualism, masculinity, uncertainty avoidance and long-term orientation. We hypothesize that countries high in these cultural dimensions are associated with more ageist health policies during the pandemic and beyond. These research ideas will extend important studies showing institutional ageism in health policies formulated by governments and international organizations (Lloyd-Sherlock et al., 2016). Future studies should test this hypothesis and design interventions for policymakers to increase their awareness and decrease implicit ageism in health policies and policy communications.

While this study circumvented the limitations of most survey studies on attitudes of individuals (e.g., Ng, Allore, & Levy, 2020; Ng, Allore, Monin, & Levy, 2016; Ng & Levy, 2018), our study is not without limitations. That the corpus compiled English sources from 20 countries is a significant limitation given the cultural differences in social construction of aging and perspectives on aging. Cruikshank (2013) emphasizes that differing geographical and cultural norms dictate how venerated aging and older adults are viewed in a society, and Kaufman (1994) argues that the concept of frailty in old adults is culturally constructed via discourses about individualism. For instance, our dataset did not include South Korea—Yun & Lachman (2006) found that Koreans have comparably higher levels of aging-related anxieties like physical appearance and younger Korean adults had greater fear of old people. In the vein, Vauclair et al. (2016) found that when comparing Taiwan to the UK, meta-perceptions about older adults were more positive (competence and admiration), but personal attitudes were more negative (prejudice and avoidance) for the former than the latter. With these complexities undergirding culture and social attitudes on aging, this is a significant limitation that will be addressed in future studies when we expand the corpus to other geographies and languages.

Another limitation is the focus on stereotypic valence (negative-positive), rather than theme. For example, while both “suffering” and “burden” may be rated negatively, the former portrays vulnerability, and the latter may signify intergenerational tensions. Future studies should investigate stereotypic themes to elucidate the nuances of ageism during the pandemic. In addition, future studies could also consider including government interventions (e.g., lockdown policies and travel restrictions), technological capabilities (e.g., Ng, 2018) and other variables such as country-level GDP, and the percentage of older adults in the population as they may influence age stereotypes.

### Conclusion

In sum, our global study of societal ageism during the Covid-19 pandemic across 20 countries found that ageism has increased significantly. This alarming acceleration of ageism deserves an urgent and concerted international effort to counteract it. The strategy to reverse this trajectory lay in the same phenomenon that promoted it: A sustained, global campaign—though, it should be culturally nuanced and customized to a country's context.

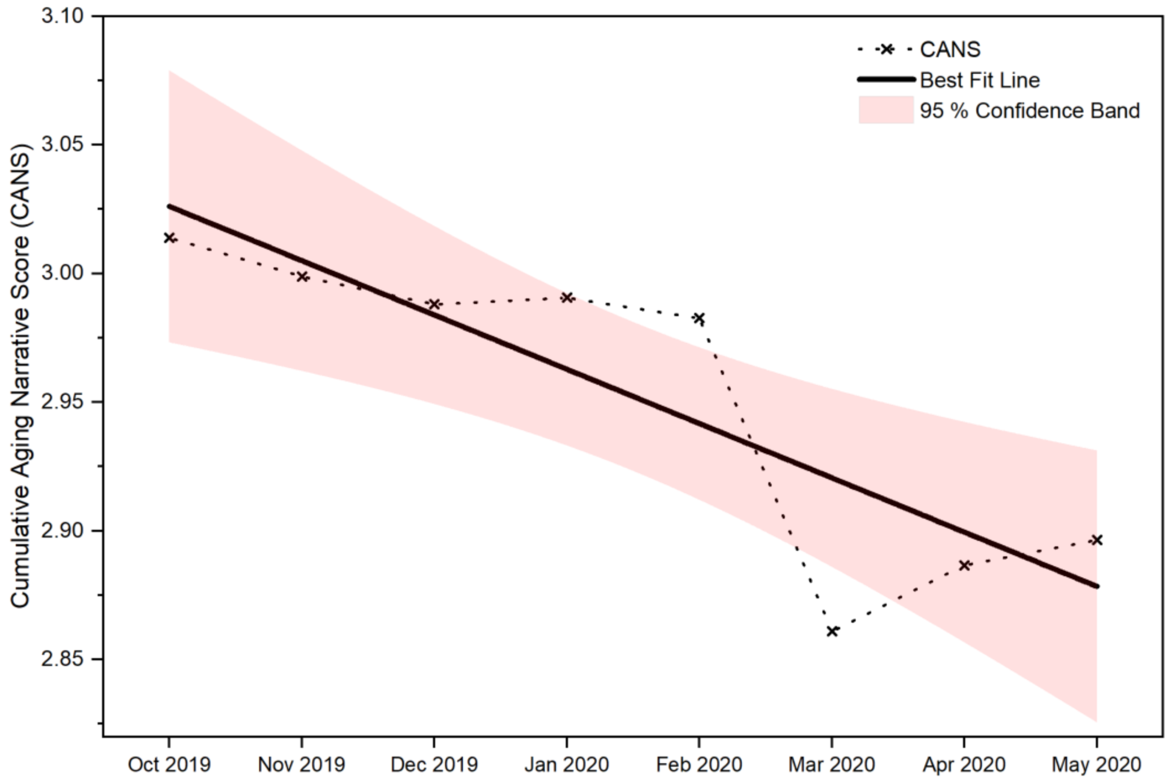

*Figure 1: Globally, aging narratives became more negative across 20 countries—a statistically significant linear trend towards negativity during the COVID-19 pandemic. Cumulative Aging Narrative Scores (CANS) were trending neutral from Oct'19 to Feb'20, and dived significantly in Mar'20. Thereafter, CANS inched upwards though it has not recovered to pre-pandemic levels.*

*Table 1. Pandemic and Cultural predictors of global aging narratives trend across 20 countries during COVID-19*

| | Model 1 | Model 2 |
|---|---|---|
| COVID-19 Incidence Rate[1] | -0.000010<br>(0.000022) | -0.000023<br>(0.000020) |
| COVID-19 Mortality Rate[2] | -0.000201<br>(0.000277) | 0.000131<br>(0.000242) |
| Individualism | | -0.001411*<br>(0.000486) |
| Masculinity | | -0.002363**<br>(0.000599) |
| Uncertainty Avoidance | | -0.002808**<br>(0.000718) |
| Long-Term Orientation | | -0.002481**<br>(0.000576) |
| Power Distance | | -0.001033<br>(0.000558) |
| N : | 160 | 144 |
| $R^2$ : | 0.2754 | 0.4645 |
| Adjusted $R^2$ : | 0.2662 | 0.4370 |

Standard errors in parentheses. ** p<0.001, * p<0.05. Constant not shown.
[1] Respective monthly COVID-19 incidence rates from Oct'19 to May'20
[2] Respective monthly COVID-19 mortality rates from Oct'19 to May'20
